# Two-Year Clinical Outcomes of Contemporary Drug-Eluting Stents Grouped by Eluted Limus Agent: A Retrospective Cohort Study

**DOI:** 10.3390/jcm15187087

**Published:** 2026-09-12

**Authors:** Donghyeon Joo, Jeong Tae Byoun, Jae Young Cho, Sungho Jo, Kyeong Ho Yun

**Affiliations:** Department of Cardiovascular Medicine, Regional Cardiocerebrovascular Center, Wonkwang University Hospital, 895 Muwang-ro, Iksan 54538, Republic of Korea; dhjoozz@naver.com (D.J.); overchaos12@gmail.com (J.T.B.); librato46@gmail.com (J.Y.C.); fuenrostu@naver.com (S.J.)

**Keywords:** drug-eluting stent, percutaneous coronary intervention, everolimus, sirolimus, zotarolimus, target vessel failure

## Abstract

**Background/Objectives:** Contemporary drug-eluting stents (DES) differ in antiproliferative drug, strut geometry, polymer, alloy, and drug release kinetics, and the independent association between the eluted drug and clinical outcomes remains uncertain. We compared outcomes among contemporary sirolimus-, zotarolimus-, and everolimus-eluting stents. **Methods:** This single-center retrospective cohort included 1940 patients who underwent percutaneous coronary intervention (PCI) between 2012 and 2021 with sirolimus- (*n* = 537), zotarolimus- (*n* = 400), or everolimus-eluting stents (*n* = 1003). The primary endpoint was target vessel failure (TVF), a composite of cardiac death, target vessel myocardial infarction (MI), or target vessel revascularization (TVR). The secondary endpoint was major adverse cardiovascular events (MACE), a composite of all-cause death, MI, or any revascularization. Stabilized inverse probability of treatment weighting (IPTW) based on pretreatment covariates and PCI era was used to address baseline and temporal differences; follow-up was censored at two years. **Results:** During a median follow-up of 731 days, weighted TVF rates were 5.4%, 7.7%, and 6.5% in the sirolimus, zotarolimus, and everolimus groups, respectively. Compared with sirolimus, TVF did not differ with zotarolimus (hazard ratio [HR] 1.38; 95% confidence interval [CI] 0.74–2.58) or everolimus (HR 1.19; 95% CI 0.70–2.02; overall *p* = 0.458). MACE also did not differ (overall *p* = 0.517). In complex PCI, TVF was higher with zotarolimus and everolimus than with sirolimus; however, event counts were small and the analyses were exploratory. **Conclusions:** In this observational cohort, no significant differences in mid-term outcomes were observed among the three drug-defined groups. Because drug type is closely intertwined with stent platform design, these findings should not be extrapolated to individual stent platforms. The exploratory complex-PCI subgroup finding requires confirmation in larger studies.

## 1. Introduction

Drug-eluting stents (DES) have become the standard device for percutaneous coronary intervention (PCI) by substantially reducing neointimal hyperplasia, restenosis, and repeat revascularization compared with bare-metal stents [1]. However, early-generation DES were associated with concerns regarding delayed arterial healing, chronic vascular inflammation, and late or very late stent thrombosis, partly attributed to thick metallic struts, durable polymer coatings, and delayed endothelialization [1,2]. These limitations led to the development of contemporary DES platforms with thinner struts, improved metallic alloys, more biocompatible or biodegradable polymers, and optimized drug release kinetics.

Most contemporary DES use the limus family of antiproliferative agents, including sirolimus, everolimus, and zotarolimus. These drugs share inhibition of the mammalian target of rapamycin pathway but differ in pharmacokinetic properties, lipophilicity, tissue retention, and release characteristics depending on the stent platform and polymer system. In parallel with these pharmacologic differences, contemporary DES platforms also vary in strut thickness, polymer type, coating distribution, radial strength, deliverability, and vascular healing profile. Therefore, in routine PCI practice, clinical outcomes after DES implantation may reflect the combined effects of the antiproliferative drug, platform design, polymer technology, patient risk, lesion complexity, and procedural factors.

Accumulating clinical evidence suggests that contemporary DES provide excellent safety and efficacy in real-world Korean PCI practice. Recent Korean prospective registries of biodegradable-polymer everolimus- or sirolimus-eluting stents have reported low rates of target vessel or device-oriented adverse outcomes, rare stent thrombosis, and favorable mid-term clinical results across all-comers, acute coronary syndrome, and complex PCI populations [3,4,5,6,7]. These findings support the high clinical performance of contemporary DES in Korean practice. Nevertheless, most previous studies have evaluated individual stent platforms or polymer technologies, and direct real-world evidence comparing clinical outcomes according to the antiproliferative drug type itself remains limited.

Clarifying whether the eluted drug type is associated with differential clinical outcomes may be clinically relevant, because multiple contemporary DES platforms are currently used interchangeably in daily PCI practice. If clinical outcomes are comparable across sirolimus-, zotarolimus-, and everolimus-eluting stents after adjustment for baseline differences, this would suggest that patient, lesion, and procedural characteristics may be more important determinants of prognosis than the antiproliferative drug category alone in the contemporary DES era. Therefore, this single-center retrospective cohort study aimed to compare clinical outcomes according to antiproliferative drug type among patients undergoing PCI with contemporary sirolimus-, zotarolimus-, or everolimus-eluting stents.

## 2. Materials and Methods

### 2.1. Study Population

**Figure 1:**
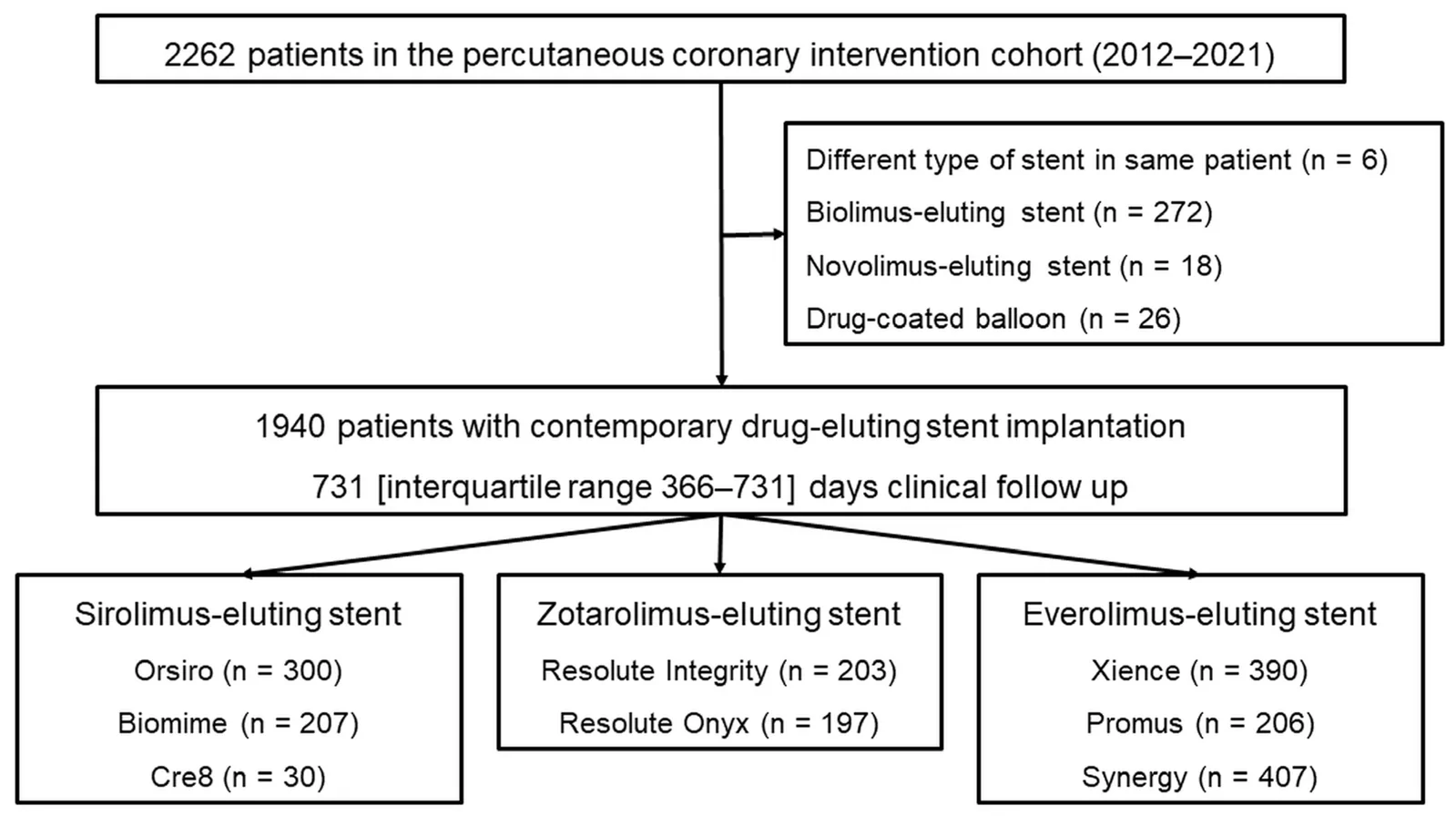
Study flow diagram.

This single-center retrospective cohort study screened 2262 patients who underwent PCI between 2012 and 2021. Patients were eligible if they underwent PCI with a contemporary DES eluting sirolimus, zotarolimus, or everolimus. Patients were excluded if they received different types of stents categorized by eluted drug during the same index procedure (*n* = 6), biolimus-eluting stents (*n* = 272), novolimus-eluting stents (*n* = 18), or drug-coated balloons (*n* = 26). Biolimus-eluting stents were excluded because all biolimus-eluting platforms implanted during the study period were stainless steel-based devices that do not represent current-generation DES, and novolimus-eluting stents were excluded because the number of treated patients (*n* = 18) was too small for an independent group analysis. Patients who received drug-coated balloons or stents of different drug categories during the same index procedure were excluded to allow unambiguous drug-level classification. After these exclusions, 1940 patients were included in the final analysis (Figure 1). The final study population was classified into three groups according to the antiproliferative drug eluted from the implanted DES. The sirolimus-eluting stent group included patients treated with Orsiro, Biomime, or Cre8 stents. The zotarolimus-eluting stent group included patients treated with Resolute Integrity or Resolute Onyx stents. The everolimus-eluting stent group included patients treated with Xience, Promus, or Synergy stents.

The study protocol was approved by the Institutional Review Board of Wonkwang University Hospital (protocol code 2026-05-025; approval date: 29 June 2026). The requirement for written informed consent was waived because of the retrospective observational design of the study.

### 2.2. Data Collection and Endpoints

Baseline clinical characteristics, angiographic findings, procedural details, laboratory results, and discharge medications were obtained from electronic medical records and the institutional PCI database. Estimated glomerular filtration rate (eGFR) was calculated using the 2021 Chronic Kidney Disease Epidemiology Collaboration creatinine equation [8]. Complex PCI was defined as the presence of at least 1 of the following procedural characteristics: treatment of ≥3 lesions, implantation of ≥3 stents, total stent length >60 mm, bifurcation PCI requiring a 2-stent technique, or PCI for chronic total occlusion [9]. Clinical follow-up was assessed from the date of the index PCI to the occurrence of a clinical event, the last available follow-up, or 731 days (2 years), whichever came first; follow-up was administratively censored at 731 days to evaluate 2-year clinical outcomes. The median follow-up duration was 731 days (interquartile range, 366–731 days). Clinical events were identified through review of electronic medical records and the institutional PCI database by study investigators. No independent blinded clinical event adjudication committee was used.

The primary endpoint was target vessel failure (TVF), defined as a composite of cardiac death, target vessel myocardial infarction (MI), or target vessel revascularization (TVR). Routine follow-up angiography was not performed, and repeat revascularization was performed only when clinically indicated for recurrent symptoms, objective evidence of ischemia, or angiographically significant restenosis or disease progression. MI was identified from the clinical diagnosis documented by the treating cardiologist together with electrocardiographic and cardiac biomarker findings available in the medical record. In accordance with the institutional database structure, only spontaneous MI was recorded, and periprocedural MI was not assessed as an endpoint. Target vessel MI was classified on the basis of coronary angiographic findings and electrocardiographic changes recorded in the database; when repeat angiography was not performed and electrocardiographic localization was ambiguous, the event was conservatively classified as target vessel MI. The secondary endpoint was major adverse cardiovascular events (MACE), defined as a composite of all-cause death, MI, or any revascularization. Additional clinical outcomes included all-cause death, cardiac death, MI, target vessel MI, TVR, non-TVR, stent thrombosis, and any revascularization [10].

### 2.3. Statistical Analysis

Continuous variables were presented as mean ± standard deviation, and categorical variables were presented as number and percentage. Baseline characteristics were compared among the sirolimus-, zotarolimus-, and everolimus-eluting stent groups in the crude population and after inverse probability of treatment weighting (IPTW). To account for differences in baseline characteristics and temporal changes in PCI practice, a multinomial propensity score model was constructed to estimate the probability of receiving each stent drug type. To avoid conditioning on post-treatment variables, the model included pretreatment covariates only: age, sex, hypertension, diabetes mellitus, current smoking, previous PCI, clinical diagnosis, hemoglobin, eGFR, LDL cholesterol, multivessel disease, calcification, chronic total occlusion, bifurcation, and PCI era. PCI era was categorized as 2012–2015, 2016–2018, and 2019–2021. Procedural characteristics and discharge medications were regarded as post-treatment (mediator) variables and were intentionally excluded from the propensity score model. Missing laboratory values (hemoglobin, *n* = 4; creatinine, *n* = 8; LDL cholesterol, *n* = 54) were imputed with the overall median for propensity score estimation. Stabilized IPTW was then applied to generate a weighted population, and weight diagnostics, including the maximum stabilized weight and the effective sample size, were examined. Covariate balance before and after IPTW was assessed using standardized mean differences (SMDs). Because there were three treatment groups, pairwise SMDs were calculated for each covariate, and the largest pairwise value was reported as the maximal SMD.

Clinical outcomes were first summarized in the crude population and then in the IPTW population. In the crude population, event rates were presented as numbers and percentages. In the IPTW population, outcomes were presented as weighted number and weighted percentage. Time-to-event analyses were performed using Cox proportional hazards regression models, with sirolimus-eluting stents as the reference group. For the crude population, unadjusted Cox proportional hazards models were used. For the IPTW population, weighted Cox proportional hazards models with robust (sandwich) standard errors were used. Hazard ratios (HRs) with 95% confidence intervals (CIs) were calculated for zotarolimus- versus sirolimus-eluting stents and everolimus- versus sirolimus-eluting stents. Overall *p* values for the 3-group comparison were obtained using 2-degree-of-freedom Wald tests. The cumulative incidence of target vessel failure (TVF) in the IPTW population was estimated using weighted Kaplan–Meier methods; the overall group difference was tested with the 2-degree-of-freedom robust Wald test from the IPTW-weighted Cox model. The number-at-risk table displays crude patient counts at each time point.

Prespecified subgroup analyses were performed for TVF according to age (<65 vs. ≥65 years), diabetes mellitus, myocardial infarction presentation, eGFR (<60 vs. ≥60 mL/min/1.73 m^2^), multivessel disease, minimal stent diameter (≤2.75 vs. >2.75 mm), and complex PCI. Subgroup-specific HRs were estimated using IPTW-weighted Cox proportional hazards models, with sirolimus-eluting stents as the reference group. *p* values for interaction were calculated by including interaction terms between stent drug type and each subgroup variable in the IPTW-weighted Cox models. These subgroup analyses were considered exploratory and were not adjusted for multiplicity. Prespecified sensitivity analyses were performed to assess the robustness of the primary analysis: (1) stabilized weights truncated at the 1st and 99th percentiles; (2) generalized overlap weighting, which targets the overlap population; (3) a doubly robust analysis in which the weighted Cox model was additionally adjusted for covariates with an absolute standardized mean difference > 0.10 after weighting; and (4) a restricted cohort of patients treated in 2016–2020, the period during which all three stent groups were implanted in every calendar year, with the propensity score model re-estimated using calendar year as a categorical variable.

As an additional descriptive analysis, crude clinical outcomes were summarized according to individual stent product. Because several product subgroups were small and product use was strongly confounded with implantation period, no formal statistical comparisons were performed across products. All tests were two-sided, and a *p* value < 0.05 was considered statistically significant. Statistical analyses were performed using SPSS version 30.0 (IBM Corp., Armonk, NY, USA) and Python version 3.12 (Python Software Foundation, Wilmington, DE, USA) with the scikit-learn and lifelines libraries.

## 3. Results

### 3.1. Baseline Characteristics and Covariate Balance

Baseline clinical, angiographic, procedural, and medication characteristics before and after IPTW are shown in Table 1. In the crude population, the three stent drug groups were generally similar with respect to age, sex, hypertension, diabetes mellitus, renal function, and LDL cholesterol level. However, current smoking, previous PCI, diagnosis, multivessel disease, stent overlap, bifurcation, complex PCI, total stent length, discharge medications, and especially PCI era differed across groups. The most prominent imbalance was observed for PCI era. Procedures performed in 2012–2015 accounted for 6.0% of the sirolimus group, 50.7% of the zotarolimus group, and 37.1% of the everolimus group, with a maximal SMD of 1.145.

**Figure 2:**
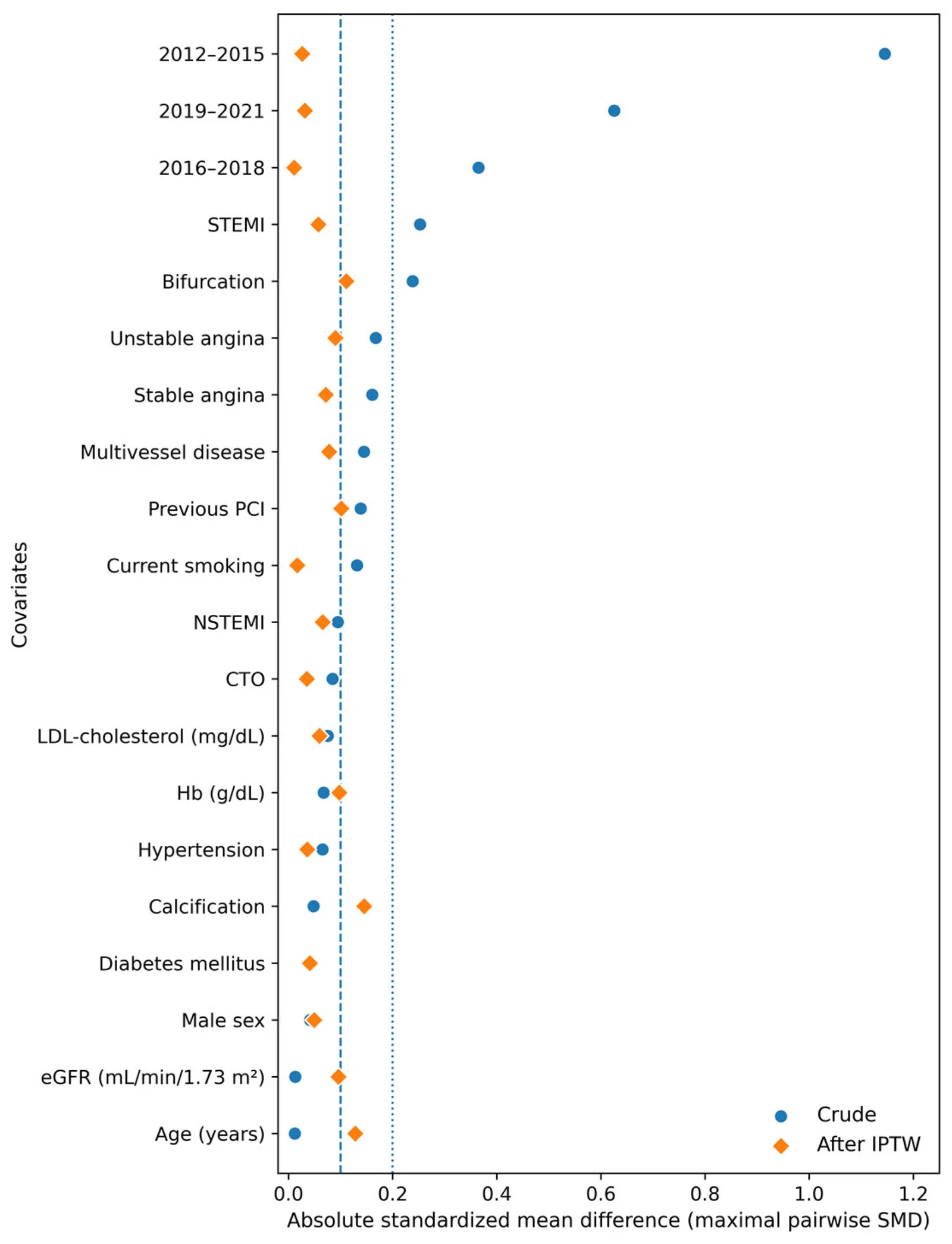
Covariate balance before and after inverse probability of treatment weighting. The Love plot shows covariate balance among the sirolimus-, zotarolimus-, and everolimus-eluting stent groups before and after stabilized inverse probability of treatment weighting. Pairwise standardized mean differences were calculated for each covariate, and the largest pairwise value was reported as the maximal standardized mean difference. The dashed and dotted vertical lines indicate maximal standardized mean differences of 0.10 and 0.20, respectively. CTO, chronic total occlusion; eGFR, estimated glomerular filtration rate; Hb, hemoglobin; IPTW, inverse probability of treatment weighting; LDL, low-density lipoprotein; NSTEMI, non-ST-segment elevation myocardial infarction; PCI, percutaneous coronary intervention; SMD, standardized mean difference; STEMI, ST-segment elevation myocardial infarction.

After IPTW, weighted patient numbers were 541.3 in the sirolimus group, 405.4 in the zotarolimus group, and 1000.6 in the everolimus group. Covariate balance improved substantially after weighting. PCI era was well balanced after weighting (maximal SMDs ≤ 0.031), and most baseline variables had maximal SMDs below 0.10 after IPTW. Residual imbalances slightly above 0.10 remained for age (0.128), previous PCI (0.102), calcification (0.145), and bifurcation (0.111); these covariates were additionally adjusted for in a doubly robust sensitivity analysis. The improvement in covariate balance before and after IPTW is summarized in Figure 2.

### 3.2. Clinical Outcomes Before and After IPTW

Clinical outcomes in the crude and IPTW populations are summarized in Table 2. In the crude population, TVF occurred in 31 patients (5.8%) in the sirolimus group, 25 patients (6.2%) in the zotarolimus group, and 63 patients (6.3%) in the everolimus group. Compared with sirolimus-eluting stents, the crude risk of TVF was not significantly different for zotarolimus-eluting stents (HR, 1.03; 95% CI, 0.61–1.74) or everolimus-eluting stents (HR, 1.05; 95% CI, 0.68–1.61; overall *p* = 0.979). No significant differences were observed in all-cause death, cardiac death, MI, target vessel MI, TVR, non-TVR, stent thrombosis, or any revascularization.

In the IPTW population, TVF occurred in 29.5 weighted patients (5.4%) in the sirolimus group, 31.1 weighted patients (7.7%) in the zotarolimus group, and 64.9 weighted patients (6.5%) in the everolimus group. Compared with sirolimus-eluting stents, the adjusted risk of TVF was not significantly different for zotarolimus-eluting stents (HR, 1.38; 95% CI, 0.74–2.58) or everolimus-eluting stents (HR, 1.19; 95% CI, 0.70–2.02; overall *p* = 0.458). The adjusted HRs for MACE were 1.22 (95% CI, 0.74–1.99) for zotarolimus versus sirolimus and 1.21 (95% CI, 0.82–1.79) for everolimus versus sirolimus (overall *p* = 0.517). The risks of all-cause death, cardiac death, MI, target vessel MI, TVR, non-TVR, stent thrombosis, and any revascularization were also not significantly different among the three groups after IPTW. The results were consistent across all prespecified sensitivity analyses; the hazard ratios for TVF and MACE remained similar in direction and magnitude, and no comparison reached statistical significance with weight truncation, generalized overlap weighting, doubly robust adjustment, or in the restricted 2016–2020 cohort (Supplementary Table S1).

**Figure 3:**
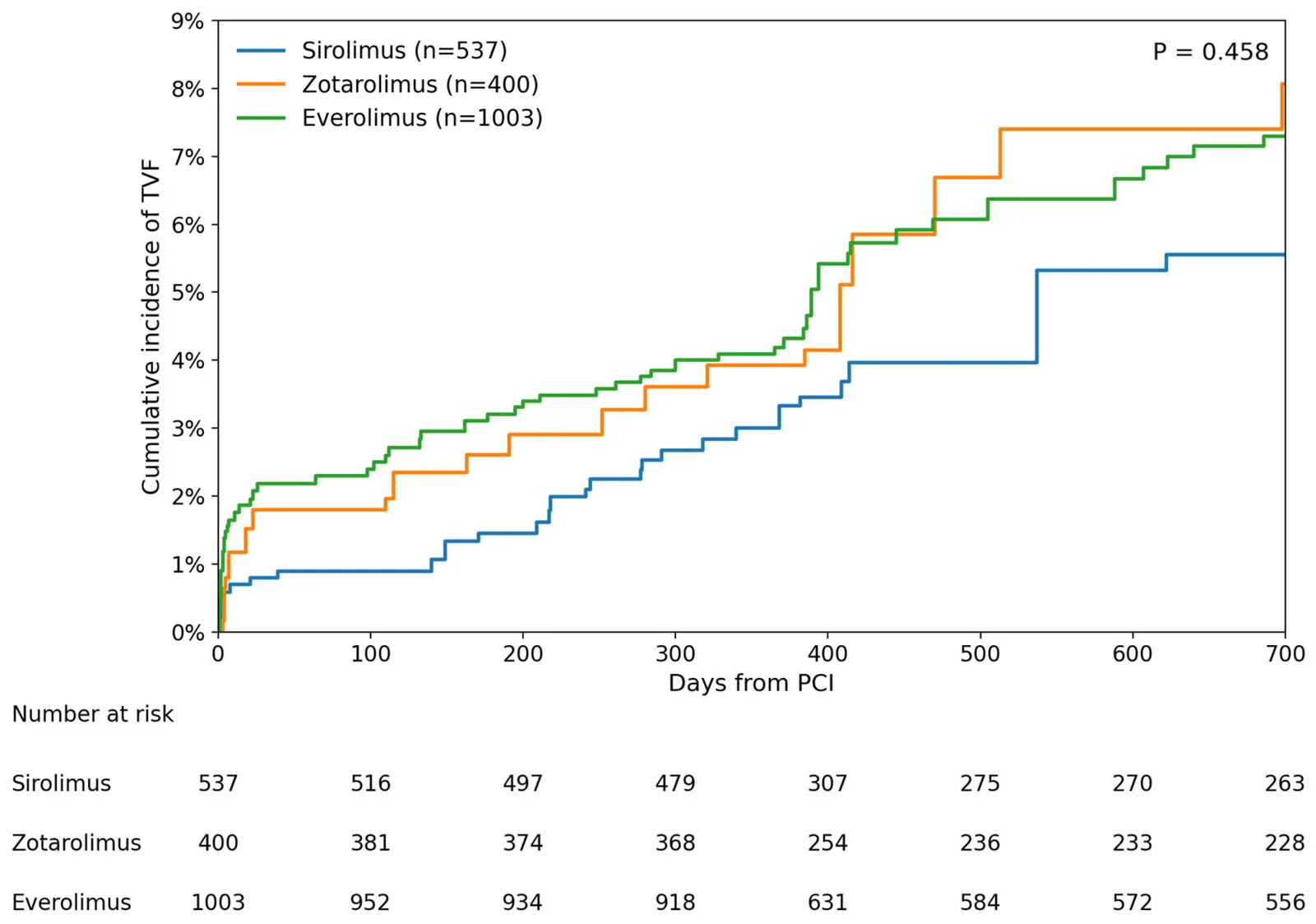
Inverse probability of treatment weighting-adjusted Kaplan–Meier curves for target vessel failure according to eluted drug type. The cumulative incidence of target vessel failure (TVF) was compared among patients treated with sirolimus-, zotarolimus-, or everolimus-eluting stents in the inverse probability of treatment weighting (IPTW)-adjusted population. TVF was defined as a composite of cardiac death, target vessel myocardial infarction, or target vessel revascularization. The numbers at risk are unweighted patient counts. Curves display events at their observed occurrence times and are truncated at 700 days. PCI, percutaneous coronary intervention.

Figure 3 shows the IPTW-adjusted Kaplan–Meier curves for TVF according to stent drug type. During two years of follow-up, the cumulative incidence curves were not significantly different among the sirolimus-, zotarolimus-, and everolimus-eluting stent groups (overall *p* = 0.458 by the two-degree-of-freedom robust Wald test from the IPTW-weighted Cox model).

### 3.3. Subgroup Analysis

**Figure 4:**
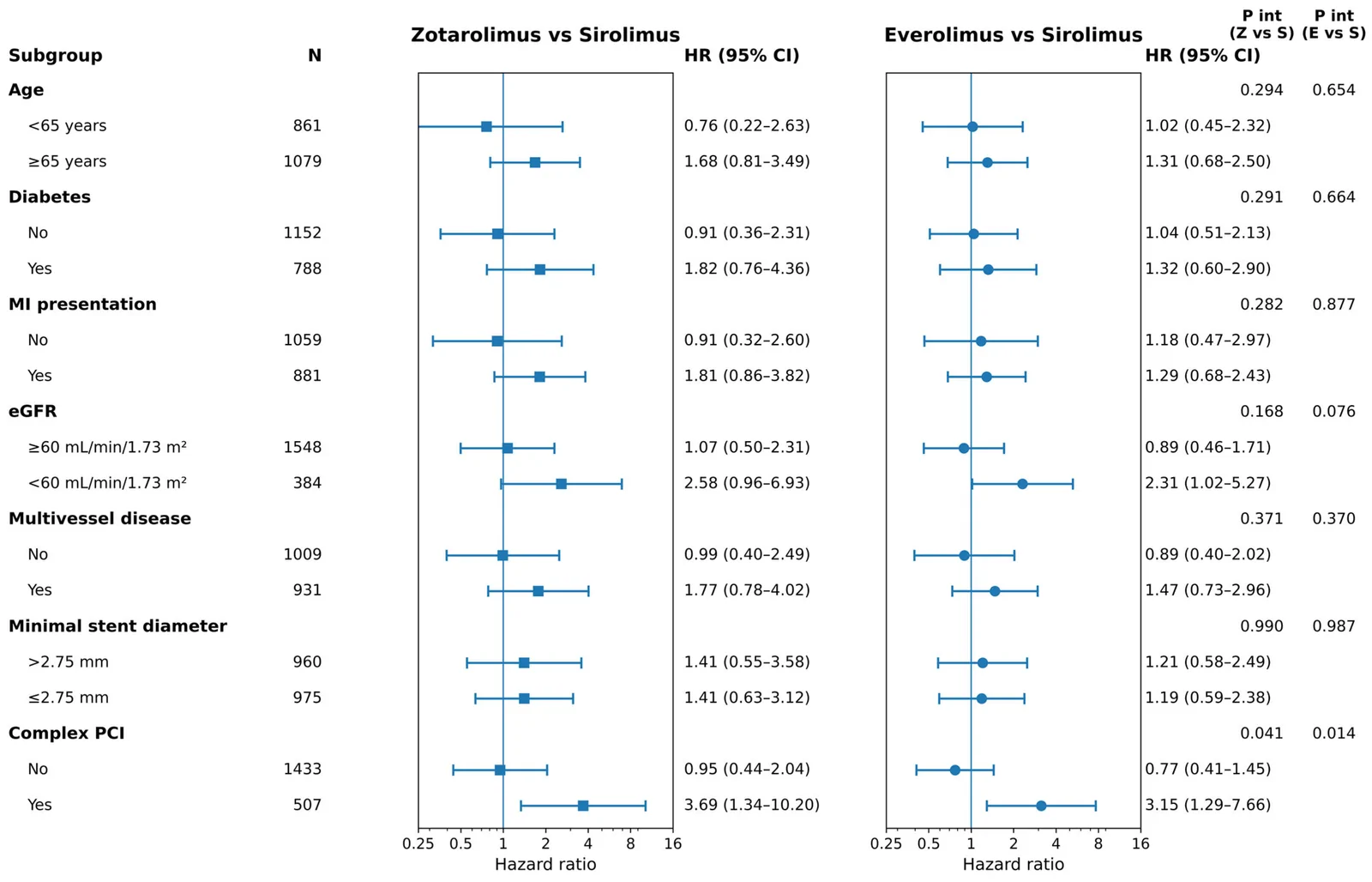
Subgroup analysis for target vessel failure according to eluted drug type. Subgroup-specific hazard ratios (HRs) and 95% confidence intervals (CIs) were estimated using inverse probability of treatment weighting (IPTW)-adjusted Cox proportional hazards models, with sirolimus-eluting stents as the reference group. The analyses were exploratory and were not adjusted for multiple testing. Squares denote hazard ratios for zotarolimus versus sirolimus and circles denote hazard ratios for everolimus versus sirolimus; the vertical line indicates a hazard ratio of 1.0. eGFR, estimated glomerular filtration rate; MI, myocardial infarction; PCI, percutaneous coronary intervention; P int, *p* value for interaction; Z vs. S, zotarolimus versus sirolimus; E vs. S, everolimus versus sirolimus.

Subgroup analyses for TVF are presented in Figure 4. In most clinically relevant subgroups, the relative risks of TVF for zotarolimus- or everolimus-eluting stents compared with sirolimus-eluting stents were directionally consistent, with no statistically significant treatment-by-subgroup interaction. There were no significant interactions according to age, diabetes mellitus, MI presentation, renal function, multivessel disease, or minimal stent diameter. In the complex PCI subgroup, the HR for TVF was 3.69 (95% CI, 1.34–10.20) for zotarolimus versus sirolimus and 3.15 (95% CI, 1.29–7.66) for everolimus versus sirolimus. The interaction *p* value was 0.041 for zotarolimus versus sirolimus and 0.014 for everolimus versus sirolimus. In contrast, among patients without complex PCI, the HRs were 0.95 (95% CI, 0.44–2.04) and 0.77 (95% CI, 0.41–1.45), respectively. These subgroup analyses were exploratory and unadjusted for multiplicity.

### 3.4. Product-Level Clinical Outcomes

Crude clinical outcomes according to individual stent product (Xience, Promus, Synergy, Orsiro, Cre8, Biomime, Resolute Integrity, and Resolute Onyx) are summarized in Supplementary Table S2. Because several product subgroups were small (e.g., Cre8, *n* = 30) and product use was strongly confounded with implantation period, these data are presented descriptively, without formal statistical comparison.

## 4. Discussion

In this single-center retrospective cohort study of patients undergoing PCI with contemporary DES, the main finding was that two-year clinical outcomes were comparable among sirolimus-, zotarolimus-, and everolimus-eluting stents after adjustment for baseline differences using IPTW. The risk of TVF, the primary endpoint of this study, did not differ significantly among the three eluted-drug groups. Similarly, MACE and individual clinical outcomes, including all-cause death, cardiac death, MI, target vessel MI, TVR, non-TVR, stent thrombosis, and any revascularization, were not significantly different. These findings suggest that, in contemporary PCI practice, the antiproliferative drug category itself may not be a major independent determinant of mid-term clinical outcomes.

The present analysis should be interpreted in the context of substantial temporal and technological changes in DES use. In crude analyses, PCI era was markedly imbalanced across the three drug groups, reflecting real-world changes in device availability and operator preference over time. Since PCI techniques, antiplatelet therapy, secondary prevention, and stent platform design have evolved over time, the calendar period can confound the association between stent type and clinical outcomes. Therefore, PCI era was included in the multinomial propensity score model. After this adjustment, covariate balance was substantially improved, and the main conclusion remained unchanged. In the revised analysis, restricting the propensity score model to pretreatment covariates further stabilized the weights and substantially narrowed the confidence intervals, without changing any conclusion. This supports the robustness of the finding that contemporary DES categorized by eluted drug type showed similar clinical performance.

Our findings are consistent with randomized comparisons of contemporary DES platforms. In the three-arm BIO-RESORT trial, TVF did not differ among ultrathin strut sirolimus-eluting, very-thin-strut everolimus-eluting, and thin-strut zotarolimus-eluting stents at three years or at the final five-year follow-up [2,11,12]. Pairwise randomized comparisons have likewise shown comparable mid- to long-term outcomes between zotarolimus- and everolimus-eluting stents in the TWENTE and DUTCH PEERS trials [13,14], and between sirolimus- and zotarolimus-eluting platforms in the BIONYX and ORIENT trials [15,16,17]. Despite differences in design, patient selection, and stent products, our real-world, drug-class-level results extend this randomized platform-level evidence to routine practice.

A key question is whether the favorable numerical trend with sirolimus-eluting stents reflects the drug itself or the devices that carried it. The sirolimus arm in our cohort consisted of Orsiro, Biomime, and Cre8, platforms characterized by ultrathin struts and, for Orsiro, a biodegradable polymer; the zotarolimus and everolimus arms were dominated by thicker-strut, durable-polymer devices. This distinction is not trivial. Bangalore et al. reported a 16% relative reduction in one-year target lesion failure with ultrathin-strut DES, driven mainly by fewer myocardial infarctions [18], and Iglesias et al. observed a comparable benefit in both chronic and acute coronary syndromes, largely through fewer clinically indicated target lesion revascularizations [19]. A network meta-analysis by Taglieri et al. placed Orsiro ahead of Xience, Resolute, and Nobori/BioMatrix for one-year target lesion failure, though the advantage faded with longer follow-up [20]. Whatever edge sirolimus-eluting stents showed in our data may therefore stem from ultrathin strut geometry or biodegradable polymer technology rather than from sirolimus itself.

Patient-level and post-procedural factors likely also contribute to mid-term outcomes beyond the eluted drug. Even after successful PCI, resting coronary blood flow in myocardial regions remote from the intervention site may remain impaired, reflecting diffuse atherosclerosis and microvascular dysfunction that are not addressed by stent selection [21]. Likewise, post-PCI management, including participation in exercise-based training after coronary stenting, may modulate the risk of in-stent restenosis and subsequent adverse events [22]. These considerations reinforce the interpretation that patient, lesion, physiological, and management factors outweigh the eluted drug category as determinants of prognosis in contemporary practice.

The same caveat applies, with added force, to the subgroup analyses. Most subgroups behaved consistently with the overall result, but in patients undergoing complex PCI the sirolimus arm fared better; the interaction reached statistical significance for both zotarolimus versus sirolimus (*p* = 0.041) and everolimus versus sirolimus (*p* = 0.014). Three considerations temper this. The subgroup tests were exploratory and unadjusted for multiplicity; the sirolimus group was disproportionately composed of newer ultrathin strut platforms such as Orsiro, which entered practice later; and outcomes in complex PCI hinge on factors we did not fully capture, from lesion calcification and bifurcation strategy to stent expansion, imaging guidance, and operator experience. The most defensible reading is not a drug-specific advantage of sirolimus but a confluence of platform design and shifting procedural practice; this is a hypothesis to be tested, not a conclusion to be drawn.

Several limitations should be acknowledged. First, this was a retrospective, single-center analysis, so residual confounding cannot be excluded even after IPTW. Second, we grouped stents by eluted drug rather than by individual platform. This mirrors how clinicians actually choose devices, but it also means the drug effect cannot be disentangled from the device-level features noted earlier, chiefly strut thickness, alloy, polymer, and release kinetics. Third, stent choice was neither randomized nor uniform over time; it tracked product availability and operator preference across a decade of practice. We entered the PCI era into the propensity model, but unmeasured secular changes likely persist. Fourth, the product-level and some subgroup comparisons rested on small numbers, particularly for the least-used stents, and are best read as descriptive. Finally, because follow-up angiography was not routine, revascularization reflects clinically driven events and undercounts silent angiographic restenosis. In addition, clinical events were identified by study investigators from electronic medical records and the institutional PCI database without an independent blinded clinical event adjudication committee, and only spontaneous MI was captured, so periprocedural MI could not be assessed. Furthermore, sirolimus-eluting stents were not implanted at our institution in 2012–2013, and zotarolimus-eluting stents were not implanted in 2021; comparisons in the earliest and latest periods therefore rely partly on model-based extrapolation, which we addressed with the restricted 2016–2020 cohort analysis.

## 5. Conclusions

After adjustment for baseline characteristics and PCI era, the two-year risk of target vessel failure did not differ significantly among contemporary sirolimus-, zotarolimus-, and everolimus-eluting stents. These observational findings do not prove the absence of a drug effect; rather, they suggest that the eluted limus agent was not a major independent determinant of mid-term outcomes relative to patient, lesion, and procedural factors, and because drug type is closely intertwined with stent platform design, they should not be extrapolated to individual stent platforms. The signal favoring sirolimus-eluting stents in complex PCI may more plausibly reflect the ultrathin-strut platforms concentrated in that group than the drug itself and should be confirmed prospectively before influencing device selection.

## Figures and Tables

**Table 1:** Baseline characteristics before and after inverse probability of treatment weighting.

	Crude Population				After IPTW			
Variables	Sirolimus (*n* = 537)	Zotarolimus (*n* = 400)	Everolimus (*n* = 1003)	Maximal SMD	Sirolimus (*n* = 541.3)	Zotarolimus (*n* = 405.4)	Everolimus (*n* = 1000.6)	Maximal SMD
Age (years)	65.9 ± 11.7	65.8 ± 11.6	65.9 ± 11.0	0.012	67.4 ± 11.5	66.3 ± 11.8	65.9 ± 11.1	0.128
Male (%)	365 (68.0)	273 (68.2)	701 (69.9)	0.042	367.1 (67.8)	271.0 (66.9)	691.9 (69.1)	0.049
Hypertension (%)	330 (61.5)	250 (62.5)	648 (64.6)	0.065	333.7 (61.6)	255.7 (63.1)	634.2 (63.4)	0.036
Diabetes mellitus (%)	226 (42.1)	161 (40.2)	401 (40.0)	0.043	210.0 (38.8)	165.4 (40.8)	405.1 (40.5)	0.041
Current smoking (%)	190 (35.4)	117 (29.2)	300 (29.9)	0.131	173.2 (32.0)	126.5 (31.2)	312.8 (31.3)	0.017
Previous PCI (%)	40 (7.4)	46 (11.5)	99 (9.9)	0.139	64.8 (12.0)	36.0 (8.9)	96.8 (9.7)	0.102
Diagnosis (%)								
Stable angina	88 (16.4)	91 (22.8)	214 (21.3)	0.161	125.8 (23.2)	82.2 (20.3)	203.1 (20.3)	0.072
Unstable angina	159 (29.6)	150 (37.5)	357 (35.6)	0.168	162.3 (30.0)	136.5 (33.7)	342.1 (34.2)	0.090
NSTEMI	154 (28.7)	98 (24.5)	261 (26.0)	0.095	159.8 (29.5)	107.7 (26.6)	267.3 (26.7)	0.066
STEMI	136 (25.3)	61 (15.2)	171 (17.0)	0.253	93.5 (17.3)	79.0 (19.5)	188.1 (18.8)	0.057
Hemoglobin (g/dL)	13.4 ± 2.1	13.5 ± 2.0	13.6 ± 2.1	0.067	13.3 ± 2.1	13.4 ± 2.0	13.5 ± 2.1	0.097
eGFR (mL/min/1.73 m^2^)	79.4 ± 26.1	79.1 ± 23.7	79.2 ± 24.8	0.012	76.7 ± 24.6	79.0 ± 24.1	79.1 ± 25.4	0.098
LDL cholesterol (mg/dL)	109.0 ± 39.5	106.0 ± 40.1	107.9 ± 42.3	0.076	107.4 ± 38.9	109.8 ± 41.5	107.6 ± 42.8	0.061
Multivessel disease (%)	230 (42.8)	199 (49.8)	502 (50.0)	0.145	279.5 (51.6)	196.6 (48.5)	477.6 (47.7)	0.078
Calcification (%)	57 (10.6)	40 (10.0)	92 (9.2)	0.048	78.3 (14.5)	39.4 (9.7)	98.5 (9.8)	0.145
CTO (%)	24 (4.5)	25 (6.2)	64 (6.4)	0.084	27.1 (5.0)	23.4 (5.8)	58.0 (5.8)	0.035
Bifurcation (%)	72 (13.4)	90 (22.5)	223 (22.2)	0.239	121.0 (22.4)	72.6 (17.9)	200.3 (20.0)	0.111
PCI era (%)								
2012–2015	32 (6.0)	203 (50.7)	372 (37.1)	1.145	173.7 (32.1)	125.1 (30.9)	313.5 (31.3)	0.026
2016–2018	278 (51.8)	136 (34.0)	370 (36.9)	0.365	218.6 (40.4)	162.9 (40.2)	407.5 (40.7)	0.011
2019–2021	227 (42.3)	61 (15.2)	261 (26.0)	0.625	149.0 (27.5)	117.3 (28.9)	279.5 (27.9)	0.031
*Post-treatment variables (not included in the propensity score model)*								
Complex PCI (%)	105 (19.6)	105 (26.2)	297 (29.6)	0.235	130.7 (24.2)	101.7 (25.1)	286.6 (28.6)	0.102
Stent number	1.5 ± 0.9	1.6 ± 0.9	1.6 ± 0.9	0.154	1.6 ± 0.9	1.6 ± 0.9	1.6 ± 0.9	0.082
Minimal stent diameter (mm)	2.9 ± 0.4	2.9 ± 0.4	2.9 ± 0.4	0.028	2.9 ± 0.4	3.0 ± 0.4	2.9 ± 0.4	0.169
Total stent length (mm)	39.9 ± 26.1	44.4 ± 28.5	45.0 ± 28.6	0.188	42.6 ± 27.4	45.4 ± 30.2	44.6 ± 28.4	0.096
Stent overlap (%)	46 (8.6)	48 (12.0)	146 (14.6)	0.188	47.7 (8.8)	54.0 (13.3)	146.2 (14.6)	0.181
Discharge medication (%)								
Aspirin	527 (98.1)	395 (98.8)	992 (98.9)	0.063	534.7 (98.8)	400.5 (98.8)	988.2 (98.8)	0.003
P2Y12 inhibitors	531 (98.9)	396 (99.0)	993 (99.0)	0.012	537.6 (99.3)	402.5 (99.3)	989.9 (98.9)	0.041
Beta blocker	331 (61.6)	287 (71.8)	718 (71.6)	0.216	351.3 (64.9)	288.8 (71.3)	706.3 (70.6)	0.137
ACEI or ARB	445 (82.9)	363 (90.8)	883 (88.0)	0.235	469.3 (86.7)	362.4 (89.4)	875.7 (87.5)	0.084
Statin	530 (98.7)	393 (98.2)	983 (98.0)	0.054	536.4 (99.1)	399.0 (98.4)	980.8 (98.0)	0.090

Values are mean ± standard deviation or number (%); IPTW values are stabilized-weighted estimates. ACEI, angiotensin-converting enzyme inhibitor; ARB, angiotensin receptor blocker; CTO, chronic total occlusion; eGFR, estimated glomerular filtration rate; LDL, low-density lipoprotein; NSTEMI, non-ST-segment elevation myocardial infarction; PCI, percutaneous coronary intervention; SMD, standardized mean difference; STEMI, ST-segment elevation myocardial infarction.

**Table 2:** Clinical outcomes before and after inverse probability of treatment weighting.

**Crude Population**						
**Outcome**	**Sirolimus (*n* = 537)**	**Zotarolimus (*n* = 400)**	**Everolimus (*n* = 1003)**	**Zotarolimus vs. Sirolimus HR (95% CI)**	**Everolimus vs. Sirolimus HR (95% CI)**	**Overall *p* Value**
All-cause death	30 (5.6)	19 (4.8)	57 (5.7)	0.81 (0.45–1.43)	0.97 (0.62–1.51)	0.737
Cardiac death	18 (3.4)	14 (3.5)	35 (3.5)	1.01 (0.50–2.03)	1.01 (0.57–1.78)	0.999
MI	17 (3.2)	8 (2.0)	31 (3.1)	0.60 (0.26–1.40)	0.94 (0.52–1.70)	0.466
Target vessel MI	8 (1.5)	5 (1.2)	24 (2.4)	0.80 (0.26–2.44)	1.54 (0.69–3.44)	0.293
TVR	15 (2.8)	13 (3.2)	27 (2.7)	1.09 (0.52–2.29)	0.92 (0.49–1.72)	0.871
Non-TVR	12 (2.2)	10 (2.5)	29 (2.9)	1.08 (0.47–2.50)	1.26 (0.64–2.46)	0.782
Any revascularization	24 (4.5)	20 (5.0)	49 (4.9)	1.06 (0.59–1.92)	1.05 (0.64–1.70)	0.977
Stent thrombosis	11 (2.0)	3 (0.8)	20 (2.0)	0.35 (0.10–1.25)	0.94 (0.45–1.96)	0.244
TVF	31 (5.8)	25 (6.2)	63 (6.3)	1.03 (0.61–1.74)	1.05 (0.68–1.61)	0.979
MACE	54 (10.1)	38 (9.5)	102 (10.2)	0.89 (0.59–1.35)	0.96 (0.69–1.34)	0.869
**IPTW Population**						
**Outcome**	**Sirolimus (*n* = 541.3)**	**Zotarolimus (*n* = 405.4)**	**Everolimus (*n* = 1000.6)**	**Zotarolimus vs. Sirolimus HR (95% CI)**	**Everolimus vs. Sirolimus HR (95% CI)**	**Overall *p* Value**
All-cause death	24.7 (4.6)	24.9 (6.1)	59.4 (5.9)	1.32 (0.68–2.56)	1.29 (0.77–2.16)	0.518
Cardiac death	15.9 (2.9)	19.2 (4.7)	37.1 (3.7)	1.59 (0.69–3.69)	1.26 (0.62–2.58)	0.388
MI	15.9 (2.9)	7.3 (1.8)	31.6 (3.2)	0.60 (0.23–1.58)	1.07 (0.52–2.22)	0.367
Target vessel MI	9.5 (1.8)	5.4 (1.3)	25.0 (2.5)	0.74 (0.20–2.78)	1.42 (0.51–3.99)	0.323
TVR	18.5 (3.4)	13.5 (3.3)	25.8 (2.6)	0.94 (0.39–2.29)	0.75 (0.34–1.64)	0.596
Non-TVR	8.1 (1.5)	7.5 (1.8)	29.6 (3.0)	1.23 (0.51–2.94)	2.00 (0.99–4.04)	0.150
Any revascularization	24.7 (4.6)	19.1 (4.7)	48.2 (4.8)	1.00 (0.49–2.05)	1.05 (0.57–1.95)	0.970
Stent thrombosis	11.2 (2.1)	4.1 (1.0)	19.7 (2.0)	0.48 (0.11–2.07)	0.95 (0.37–2.43)	0.401
TVF	29.5 (5.4)	31.1 (7.7)	64.9 (6.5)	1.38 (0.74–2.58)	1.19 (0.70–2.02)	0.458
MACE	46.1 (8.5)	43.1 (10.6)	103.8 (10.4)	1.22 (0.74–1.99)	1.21 (0.82–1.79)	0.517

Values are presented as numbers (%) in the crude population and as weighted numbers (%) in the inverse probability of treatment weighting (IPTW) population. Hazard ratios (HRs) and 95% confidence intervals (CIs) were estimated using Cox proportional hazards models with sirolimus-eluting stents as the reference group; weighted models used robust standard errors, and overall *p* values are from 2-degree-of-freedom Wald tests. Target vessel failure (TVF) was defined as a composite of cardiac death, target vessel myocardial infarction (MI), or target vessel revascularization (TVR). Major adverse cardiovascular events (MACE) were defined as a composite of all-cause death, MI, or any revascularization.

## Data Availability

The data presented in this study are available from the corresponding author upon reasonable request. The data are not publicly available because of institutional data protection regulations and patient privacy.

## Supplementary Materials

The following supporting information can be downloaded at: https://www.mdpi.com/article/10.3390/jcm15187087/s1, Table S1: Sensitivity analyses for target vessel failure and MACE at 2 years; Table S2: Two-year clinical outcomes according to individual stent product.

## Abbreviations

The following abbreviations are used in this manuscript:

ACEI	angiotensin-converting enzyme inhibitor
ARB	angiotensin receptor blocker
CI	confidence interval
CTO	chronic total occlusion
DES	drug-eluting stent
eGFR	estimated glomerular filtration rate
Hb	hemoglobin
HR	hazard ratio
IPTW	inverse probability of treatment weighting
LDL	low-density lipoprotein
MACE	major adverse cardiovascular events
NSTEMI	non-ST-segment elevation myocardial infarction
PCI	percutaneous coronary intervention
SMD	standardized mean difference
STEMI	ST-segment elevation myocardial infarction
TVF	target vessel failure
TVR	target vessel revascularization
