# Supplementary material for: Two-Year Clinical Outcomes of Contemporary Drug-Eluting Stents Grouped by Eluted Limus Agent: A Retrospective Cohort Study
Source: J Clin Med. 2026 Sep 12;15(18):7087. doi: 10.3390/jcm15187087 (PMC13607187; doi:10.3390/jcm15187087)
Supplement: Supplementary file 1 [file jcm-15-07087-s001.zip › jcm-4476363-supplementary.pdf]

Supplementary Materials

Table S1. Sensitivity analyses for target vessel failure and MACE at 2 years

Analysis	Target vessel failure			MACE			Maximum weight	Effective sample size
	ZES vs. SES, HR (95% CI)	EES vs. SES, HR (95% CI)	p value	ZES vs. SES, HR (95% CI)	EES vs. SES, HR (95% CI)	p value		
Primary analysis (stabilized IPTW)	1.38 (0.74–2.58)	1.19 (0.70–2.02)	0.458	1.22 (0.74–1.99)	1.21 (0.82–1.79)	0.517	13.7	1263
Weights truncated at 1st/99th percentiles	1.24 (0.67–2.30)	1.07 (0.63–1.79)	0.681	1.09 (0.67–1.77)	1.08 (0.74–1.58)	0.886	4.0	1562
Generalized overlap weighting	1.39 (0.77–2.49)	1.15 (0.71–1.85)	0.773	1.14 (0.71–1.81)	1.10 (0.77–1.57)	0.942	0.9	1204
Doubly robust adjustment†	1.47 (0.77–2.80)	1.30 (0.75–2.26)	0.314	1.32 (0.79–2.19)	1.34 (0.89–2.02)	0.239	13.7	1263
Restricted cohort, 2016–2020‡	1.51 (0.82–2.78)	1.26 (0.78–2.03)	0.373	1.02 (0.59–1.75)	1.08 (0.75–1.56)	0.907	2.9	1170

Hazard ratios (HRs) and 95% confidence intervals (CIs) were estimated using weighted Cox proportional hazards models with robust standard errors, with the sirolimus-eluting stent group as the reference; p values are from 2-degree-of-freedom robust Wald tests of the overall group effect. In the primary analysis, stabilized inverse probability of treatment weights were derived from a multinomial logistic propensity score model including age, sex, hypertension, diabetes mellitus, current smoking, previous PCI, clinical diagnosis, hemoglobin, estimated glomerular filtration rate, LDL-cholesterol, multivessel disease, calcification, chronic total occlusion, bifurcation, and PCI era (2012–2015, 2016–2018, 2019–2021). Generalized overlap weighting targets the overlap population. Effective sample size was calculated as $(\Sigma w)^2/\Sigma w^2$.

† Weighted Cox model additionally adjusted for age, previous PCI, calcification, and bifurcation (covariates with an absolute standardized mean difference > 0.10 after weighting).

‡ Patients treated in 2016–2020, the period during which all three stent groups were implanted in every calendar year (n = 1,297; sirolimus 474, zotarolimus 197, everolimus 626); the propensity score model was re-estimated within this cohort with calendar year included as a categorical variable.

CI, confidence interval; EES, everolimus-eluting stent; HR, hazard ratio; IPTW, inverse probability of treatment weighting; MACE, major adverse cardiovascular events; PCI, percutaneous coronary intervention; SES, sirolimus-eluting stent; ZES, zotarolimus-eluting stent.

Table S2. Two-year clinical outcomes according to individual stent product (descriptive)

Outcome	Xience (n = 390)	Promus (n = 206)	Synergy (n = 407)	Orsiro (n = 300)	Cre8 (n = 30)	Biomime (n = 207)	Resolute Integrity (n = 203)	Resolute Onyx (n = 197)
All-cause death	20 (5.1)	13 (6.3)	24 (5.9)	13 (4.3)	2 (6.7)	15 (7.2)	5 (2.5)	14 (7.1)
Cardiac death	13 (3.3)	9 (4.4)	13 (3.2)	9 (3.0)	1 (3.3)	8 (3.9)	3 (1.5)	11 (5.6)
MI	11 (2.8)	10 (4.9)	10 (2.5)	9 (3.0)	0 (0.0)	8 (3.9)	5 (2.5)	3 (1.5)
Target vessel MI	8 (2.1)	7 (3.4)	9 (2.2)	6 (2.0)	0 (0.0)	2 (1.0)	2 (1.0)	3 (1.5)
TVR	8 (2.1)	6 (2.9)	13 (3.2)	9 (3.0)	0 (0.0)	6 (2.9)	6 (3.0)	7 (3.6)
Non-TVR	9 (2.3)	8 (3.9)	12 (2.9)	7 (2.3)	0 (0.0)	5 (2.4)	8 (3.9)	2 (1.0)
Any revascularization	14 (3.6)	14 (6.8)	21 (5.2)	15 (5.0)	0 (0.0)	9 (4.3)	11 (5.4)	9 (4.6)
TVF	22 (5.6)	14 (6.8)	27 (6.6)	17 (5.7)	1 (3.3)	13 (6.3)	8 (3.9)	17 (8.6)
MACE	34 (8.7)	25 (12.1)	43 (10.6)	29 (9.7)	2 (6.7)	23 (11.1)	16 (7.9)	22 (11.2)

Values are number of patients (%) based on crude (unweighted) 2-year event counts. Because several product subgroups were small (e.g., Cre8, n = 30) and product use was strongly confounded with implantation period, no formal statistical comparisons were performed; these data are presented for descriptive purposes only.

Target vessel failure (TVF) was defined as a composite of cardiac death, target-vessel myocardial infarction (MI), or target vessel revascularization (TVR). Major adverse cardiovascular events (MACE) were defined as a composite of all-cause death, MI, or any revascularization. Non-TVR denotes revascularization of a non-target vessel.

MACE, major adverse cardiovascular events; MI, myocardial infarction; TVF, target vessel failure; TVR, target vessel revascularization.
